# Draft genome sequence of *Bacillus anthracis* TIg03 isolated from agricultural soil in Morocco

**DOI:** 10.1128/mra.01453-25

**Published:** 2026-04-20

**Authors:** Houda Zouagui, Rahma Zouagui, Laila Sbabou

**Affiliations:** 1 Microbiology and Molecular Biology Team, Center of Plant and Microbial Biotechnology, Biodiversity and Environment, Faculty of Sciences, Mohammed V University in Rabat 107736 , Rabat, Morocco; Fluxus Inc., Sunnyvale, California, USA

**Keywords:** *Bacillus anthracis*, environment, whole genome, wgSNP analysis

## Abstract

We report the draft genome sequence of *Bacillus anthracis* TIg03, isolated from agricultural soil in southwest Morocco. Strain TIg03 lacks the pXO1 and pXO2 plasmids carrying key anthrax virulence genes. wgSNP phylogeny placed TIg03 among typical environmental *B. anthracis* strains.

## ANNOUNCEMENT

*Bacillus anthracis* is gram-positive, spore-forming, non-motile, non-hemolytic bacterium of the *Bacillus cereus* group (1), causes anthrax and forms spores that persist in soil for decades (2, 3). *B. anthracis* virulence is mediated by two plasmids: pXO1, encoding anthrax toxin genes (4), and pXO2, carrying capsule synthesis gene clusters (5). Compared to highly virulent clinical isolates, environmental strains may exhibit reduced pathogenicity and distinct genetic features (6–11).

*B. anthracis* TIg03 was isolated from agricultural soil collected in the village of Ignaren (30°49′5.14″ N, 7°86′5.25″ W), Morocco. Soil samples were suspended in sterile 0.9% (wt/vol) saline and serially diluted. Aliquots of 100 μL from each dilution were plated onto Luria-Bertani (LB) agar (12) and incubated at 28°C for 48 h. Colonies were purified by repeated streaking and stored in 40% glycerol at –80°C. Genomic DNA was extracted from a 24-h LB liquid culture grown at 28°C under shaking conditions, using the PureLink Genomic DNA Kit (Invitrogen, Thermo Fisher Scientific), following the manufacturer’s instructions. Extracted DNA was quantified using a Qubit 4.0 fluorometer and assessed on a 0.8% agarose gel. Sequencing library was prepared using the Rapid Barcoding Kit (SQK-RBK004) and sequenced on a GridION device (Oxford Nanopore Technologies, Oxford, UK) using a FLO-MIN106D (R9.4.1) flow cell for 72 h. Sequencing data were then basecalled using Guppy (v5.0.7) (13). Raw reads were adapter-trimmed using Porechop (v0.2.4) (14) and *de novo* assembled with Canu (v2.2) (15). Contigs were subsequently polished using Racon (v1.5.0) (16), Medaka (v2.0.1) (https://github.com/nanoporetech/medaka), and Homopolish (v0.3.4) (17). Strain identification was carried out by ribosomal protein subunit (*rps*) typing using the Public Databases for Molecular Typing and Microbial Genome Diversity (PubMLST) server (https://pubmlst.org/) (18). Identification was further confirmed by average nucleotide identity (ANI) using pyANI (v0.2.9) (19) and digital DNA–DNA hybridization (dDDH) using the Genome-to-Genome Distance Calculator 3.0 (GGDC) server (20), against validly described type strains of the *Bacillus cereus* group. Genome annotation was performed using the NCBI Prokaryotic Genome Annotation Pipeline (PGAP) (v.6.10) (21) and the PubMLST Genome Comparator tool (18, 22). TIg03 and 11 clinical and environmental *B. anthracis* strains were analyzed by whole-genome single nucleotide polymorphism (wgSNP) using CSI Phylogeny (v1.4) (23), and SNP-based phylogeny with an altered FastTree (v2.1.8) (24). Default parameters were applied unless specified. The assembled genome has a size of 5.3 Mb across two contigs. Sequencing and assembly statistics are presented in Table 1. *rps* typing identified TIg03 as *B. anthracis* with 100% prediction support. TIg03 exhibited the highest ANI and dDDH of 97.71% and 78.20%, respectively, with *B. anthracis* type strain (GCF_022221345.1).

**TABLE 1:** Sequencing data and genome assembly statistics of strain TIg03

Parameter	TIg03
Number of raw reads	175,056
Read *N*_50_	7,587 bp
Genome coverage	270×
Assembled length	5,332,730 bp
Number of contigs	2
Contig *N*_50_	5,239,434 bp
Assembly coverage	88.13%
Completeness	95.9%
Contamination	2.6%
GC content	35.5%
CDSs^*a*^	5,589
rRNA^*a*^	42
tRNA^*a*^	105
ncRNA^*a*^	2

^*a*^ CDSs, coding sequences; ncRNA, non-coding RNA; rRNA, ribosomal RNA; tRNA, transfer RNA.

**Fig 1:**
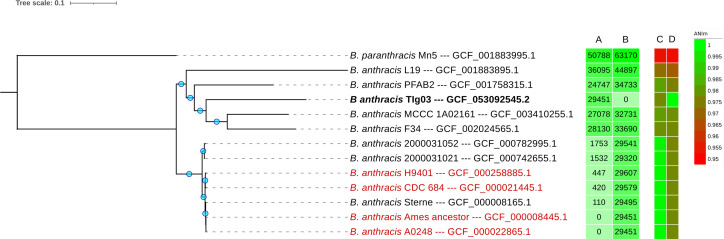
Approximately maximum likelihood wgSNP Tree of TIg03 and 11 *B. anthracis* strains from clinical and environmental sources. Strains used for the analysis: L19 (GCF_001883895.1) and MCCC 1A02161 (GCF_003410255.1) from sea sediment in China; PFAB2 (GCF_001758315.1) from hot water spring in India; F34 (GCF_002024565.1) from salty lake in Algeria; 2000031052 (GCF_000782995.1) from anthrax-diseased *Bos taurus* in USA; 2000031021 (GCF_000742655.1) from soil in USA; Sterne (GCF_000008165.1) a laboratory-derived strain used for vaccine; H9401 (GCF_000258885.1) from a cutaneous anthrax patient in South Korea; CDC 684 (GCF_000021445.1) a naturally occurring, avirulent strain, carrying both virulence plasmids; A0248 (GCF_000022865.1) a human isolated strain; Ames ancestor (GCF_000008445.1) the reference strain of virulent *B. anthracis*; and *B. paranthracis* Mn5 (GCF_001883995.1) as outgroup. Strains indicated in red are carrying both plasmids pXO1 and pXO2. Blue circles on the branches indicate local-support values of at least 94% using 1,000 resamples. (**A**) and (**B**) panels display pairwise SNPs against Ames ancestor and TIg03 strains, respectively. (**C**) and (**D**) panels show ANI values against the type strain *B. anthracis* Vollum (GCF_022221345.1) and TIg03 strains, respectively. TIg03 clustered with environmental strains lacking one or both pXO plasmids and showing 97–98% ANI to the *B. anthracis* type strain and to each other, with high pairwise SNPs (>29,000) relative to both environmental and clinical strains. Virulent strains formed a distinct clade with ≥99% ANI and low pairwise SNPs (<1,800) among typical *B. anthracis* strains. *B. anthracis* TIg03 displays an environmental strain profile.

Sequencing reads were mapped to reference pXO1 and pXO2 plasmid sequences, and mapping-guided re-assembly revealed no plasmid-like fragments. Manual and alignment-based functional analysis of annotated proteins corroborates the absence of pXO1- and pXO2-encoded anthrax virulence genes, including those encoding the edema factor (*cya*), lethal factor (*lef*), and the protective antigen (*pagA*), as well as the capsule synthesis gene clusters *capABCDE* and *acpAB*, which mediate resistance to phagocytosis and systemic dissemination (25, 26). The wgSNP analysis further supports the environmental characterization of TIg03 (Fig. 1).

## Data Availability

The draft genome assembly of Bacillus anthracis TIg03 is available under the DDBJ/ENA/GenBank accession number NZ_JBROTA000000000.2, with BioSample number SAMN50141656, and BioProject number PRJNA827450. The raw reads are deposited in the NCBI Sequence Read Archive (SRA) under accession number SRX29993468.
